# The Emergence of Environmental Health Literacy—From Its Roots to Its Future Potential

**DOI:** 10.1289/ehp.1409337

**Published:** 2015-06-30

**Authors:** Symma Finn, Liam O’Fallon

**Affiliations:** Division of Extramural Research and Training, National Institute of Environmental Health Sciences, National Institutes of Health, Department of Health and Human Resources, Research Triangle Park, North Carolina, USA

## Abstract

**Background:**

Environmental health literacy (EHL) is coalescing into a new subdiscipline that combines key principles and procedural elements from the fields of risk communication, health literacy, environmental health sciences (EHS), communications research, and safety culture. These disciplines have contributed unique expertise and perspectives to the development of EHL. Since 1992, the National Institute of Environmental Health Sciences (NIEHS) has contributed to the evolution of EHL and now seeks to stimulate its scientific advancement and rigor.

**Objectives:**

The principal objective of this article is to stimulate a conversation on, and advance research in, EHL.

**Discussion:**

In this article, we propose a definition of and conceptual framework for EHL, describe EHL in its social and historical context, identify the complementary fields and domains where EHL is being defined and implemented, and outline a research agenda. Extensive reviews of web and literature searches indicate that the concept of EHL is evolving rapidly, as are the definitions of its scope and inquiry. Although several authors have outlined different frameworks, we believe that a more nuanced model based on Bloom’s taxonomy is better suited to EHL and to future research in this area.

**Conclusions:**

We posit that EHL can potentially benefit the conduct and outcomes of community-engaged and health disparities EHS research and can ensure that the translation of research findings will lead to greater understanding of specific risks, reduction of exposures, and improvement of health outcomes for individuals and communities. We provide four recommendations to advance work in EHL.

**Citation:**

Finn S, O’Fallon L. 2017. The emergence of environmental health literacy—from its roots to its future potential. Environ Health Perspect 125:495–501; http://dx.doi.org/10.1289/ehp.1409337

## Defining the Scope and Purpose of Environmental Health Literacy (EHL)

Fundamentally, environmental health literacy (EHL) begins with an understanding of the link between environmental exposures and health. EHL has recently coalesced as a new subdiscipline combining key principles and procedural elements from the fields of health literacy, risk communication, environmental health sciences (EHS), communications research, and safety culture (Biocca 2004; Chinn 2011; Edwards et al. 2013; Fitzpatrick-Lewis et al. 2010; Nicholson 2000). Each of these disciplines has contributed unique frameworks and perspectives to the development of EHL as a distinct subfield and is likely to continue to inform the evolution of EHL.

The purpose of this article is to propose a definition of and a conceptual framework for EHL, to understand EHL in its social and historical contexts, and to identify the complementary fields and domains where EHL is being defined and implemented. This commentary acknowledges the value of current academic efforts to delineate the progressive nature of EHL that begins with an individual’s understanding and proceeds to the ability to create new information because similar to health literacy, EHL is not a static achievement, but an evolutionary process.

Another purpose of this article is to highlight the role that the National Institute of Environmental Health Sciences (NIEHS) has played in advancing the concept of EHL and to outline a research agenda that will move forward and stimulate the development of research on this topic. Similar to the validated benefits health literacy can provide in biomedical settings (Benjamin 2010; Lin et al. 2004), we propose that EHL can potentially benefit the conduct and outcomes of community-engaged and health disparities environmental health sciences (EHS) research as well as efforts to promote environmental justice. We also propose that EHL can ensure that the translation of research findings leads to a greater understanding of specific risks, reduction of exposures, and improvement of health outcomes for individuals and communities.

Our extensive literature searches of PubMed (http://www.ncbi.nlm.nih.gov/pubmed) and Web of Science (http://apps.webofknowledge.com) confirm that the field is evolving rapidly, as are definitions of the scope of inquiry and purpose of EHL. Academic endeavors to date have focused primarily on elucidating the attributes of EHL and on the stages of becoming literate about environmental health concepts and issues (Kaphingst et al. 2012; Sørensen et al. 2012). These academic efforts have built upon conceptual frameworks from the fields of health literacy and risk communication to define the progression of understanding from basic knowledge to comprehension and application (Colucci-Gray et al. 2006; Guidotti 2013; Nutbeam 2008). Addressing gaps in education and promoting EHL among health care professionals via curricula and educational module development is another major theme that emerged from the literature review (Barnes et al. 2010; Gehle et al. 2011).

A review of the existing literature related to EHL makes it clear, however, that raising EHL is more than simply the stages of an educational process. It also represents a philosophical perspective, a public health policy to improve literacy and health literacy in the general public, and a set of strategies to empower individuals and communities to exert control over the environmental exposures that may lead to adverse health outcomes (Estacio 2013; Minkler et al. 2008; Mogford et al. 2011; Zoller 2012).

Environmental health literacy integrates concepts from both environmental literacy and health literacy to develop the wide range of skills and competencies that people need in order to seek out, comprehend, evaluate, and use environmental health information to make informed choices, reduce health risks, improve quality of life and protect the environment. (Society for Public Health Education; http://www.sophe.org/environmentalhealth/key_ehl.asp)

Existing definitions of EHL, such as the one that the Society for Public Health Education (SOPHE) first outlined in 2008, often include language connoting the evolutionary nature and stages of EHL (Hatfield 1994; Nutbeam 2009); however, we propose a baseline definition that emphasizes the underlying issue: an understanding of the connection between environmental exposures and human health. As we discuss later, this understanding is only the first stage of a hierarchy of increasing literacy. We believe that this baseline definition enables EHL to be described through related disciplinary perspectives such as health literacy, risk communication, EHS, communications, public health, and the social sciences. As EHL evolves, it will be measured and applied in many ways depending on the disciplinary lens, the aim, and the audience.

## The Historical Roots of Environmental Health Literacy

**Figure 1:**
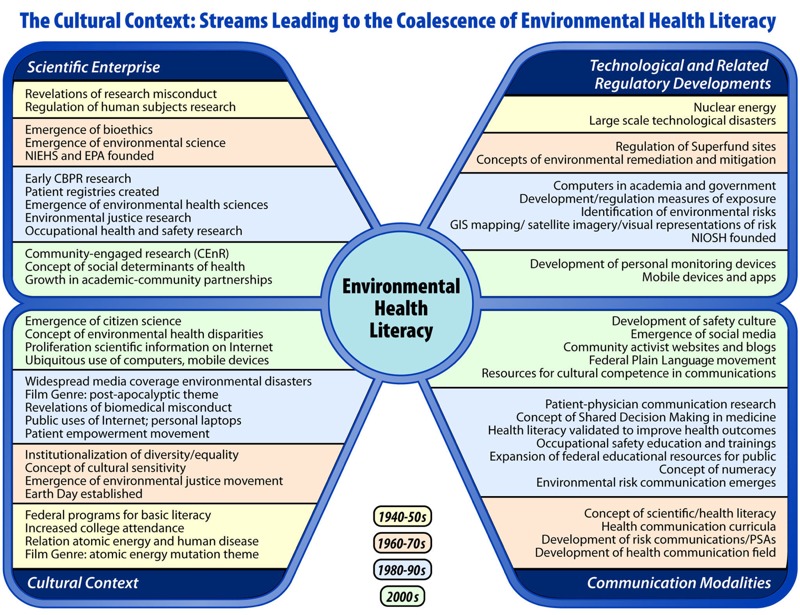
The cultural context: streams leading to the coalescence of environmental health literacy.

There are a number of different sources of the emergence of environmental health literacy (Figure 1). Risk communication, one of EHL’s roots, has deep historical origins and can be traced to the display of symbols in ancient cultures to connote tribal and state affiliations on the battlefield. More recent historical examples of risk communication also utilized symbols to connote danger: the well-known skull and crossbones symbol used initially by pirates and then later as the symbol for poison, and the color red that is widely used to indicate “stop” or “danger” (Hancock et al. 2004). World War II expanded the symbolic vocabulary for dangerous and toxic situations, and the postwar era adopted much of this military iconography in high-risk and dangerous settings related to toxic chemicals, imminent danger, poison, and, increasingly in the 1950s, as symbols for nuclear energy’s threat (Matthews et al. 2014; Young 1998). Symbolic representations are recognized as an effective and appropriate method of communicating hazards; however, cultural differences in risk perception and in the interpretation of specific colors or icons has led to the consideration of universal symbols and to research evaluating the optimal formats for communicating environmental risks (Chan and Ng 2012; Lesch et al. 2009).

More recent impetus for the development of EHL began in the late 20th century with the recognition that risks to human health came from a number of different environmental sources and had varying levels of immediate toxicity that could not be sufficiently communicated via an icon or symbol (Hancock et al. 2004). This understanding of complex risk was encapsulated in the 1960s with the publication of *Silent Spring* (Carson et al. 1962) and was further elucidated by Rachel Carson’s testimony to Congress on pesticides in 1963. Although much of the data that she presented was known to the scientific community, Carson was the first to explain to policy makers and the general public the far-reaching consequences of the introduction of chemicals into the environment in such compelling and convincing terms. Through her vision of a compromised environment, “Carson, the citizen–scientist, spawned a revolution” (Griswold 2012) that led to the rise of organized environmental activism.

Concurrent with this new societal awareness of environmental risks, the NIEHS and the U.S. Environmental Protection Agency (EPA) were established (1966 and 1970, respectively), and early efforts to explore environmental sciences expanded into consideration of the effects of pollutants and other environmental exposures on human health. Since 1970, EHL has been coalescing as a distinct field in direct proportion to the federal commitment to provide information to the public, including EHS research findings, and to the increased public awareness of environmental risks.

Articles describing the historical basis for the emergence of EHL often point to its roots in the health literacy movement in the United States. However, EHL is more than an extension of health literacy, it is the logical and inevitable outcome of the validation of health literacy to improve health outcomes and treatment adherence (Benjamin 2010; Paasche-Orlow et al. 2005) and the extrapolation of that value to the prevention of environmentally induced disease. The coalescence of EHL as a distinct subfield may also be attributed to the recognition of the public health implications of environmental health research with affected communities (Brown et al. 2012; Perez et al. 2012) and the need for research to identify and address environmental risks. Recent reports show that health literacy efforts have evolved, and these reports indicate recognition of the need to move beyond the health care setting and system [Institute of Medicine of the National Academies (IOM) 2004, 2011]. EHL acknowledges this need and addresses the health context of the individual and the community. The goals of EHL are consistently focused on preventing illness by raising awareness of risks from environmental factors and by providing approaches that individuals and communities can take to avoid, mitigate, or reduce such exposures.

The cultural shift in the value of scientific literacy among the general public also stimulated the evolution of the concept of EHL. Analogous to the rise of bioethics in the context of genetics research, EHL arose in response to growing public interest in the environment as well as to scientific and technological advancements that were increasingly available to the public. Furthermore, the emergence of the Environmental Justice movement drew political attention to inequitable and disproportionate environmental exposures faced by low-income, minority, and indigenous populations (Stokes et al. 2010). These and other concerns about environmental pollutants in air, food, and water also led to the emergence of citizen science and the necessity for health risk communications related to environmental exposures (Bonney et al. 2014; Conrad and Hilchey 2011; Minkler et al. 2010).

Scientific and technological developments also contributed to the evolution of communication modalities related to environmental risk that are not dependent on reading ability. In this context, the emergence of EHL can be considered the next stage in risk assessment and a reflection of advances in the fields of exposure assessment and exposure biology. In the 1980s and 1990s, technologies were developed to measure environmental toxicants, standards and regulations were established for chemical exposure and “levels of concern,” and there was an increase in the availability of computer-based visual representations of risk. With the widespread adoption of computers in the 1990s and the development of geographic information system (GIS) mapping software, computer-based visual representations of risk and the ability to link relative risk to geographic locations emerged as an accessible and cost-effective communication modality for the public (Lahr and Kooistra 2010; Severtson 2013). The field of risk communication was an early adopter of visual representations of risk. Such communications represented the most rapid means of translating evidence into risk messages and offered a modality that was both understandable and meaningful for individuals with varying levels of basic and scientific literacy (Hermer and Hunt 1996; LePrevost et al. 2013).

The roots of EHL can also be traced to widespread public awareness of human-made technological disasters that caused large-scale environmental pollution (Brennan 2009). Since the 1980s, media attention to such accidents has been so extensive that one need only mention the Bhopal chemical spill, Love Canal, the Three Mile Island, Chernobyl, or Fukushima nuclear accidents, or the *Exxon Valdez* or *Deepwater Horizon* oil spills to elicit images of severe and pervasive contamination. The impact of these disasters was communicated by newspaper photos of oil-soaked marine birds or workers in HazMat suits, televised images of billowing clouds of oil gushing from the wellhead, or YouTube videos of tar balls on the beach. Public attention to such extreme polluting events is heightened by the ever-increasing amounts of information on the Internet about the negative health impacts of the multiple exposures we all experience throughout our lives (Murphy et al. 2010).

## The Social Context Underlying the Development of EHL

Although several authors recognize the various roots that have come together and flowered into the emergence of EHL (Baur 2010; Huber et al. 2012), there is little in the literature that explores the larger cultural context that underlies how the public understands environmental health risks. As efforts are made to promote the value of EHL, it will be important to comprehend and address public understanding and misunderstanding of environmental risks and how this knowledge has been informed and defined by cultural media (i.e., books, films, television) (Frayling 2005; Kennedy et al. 2011; Moore 2015; Murphy et al. 2010).

Films have historically explored and exploited public awareness of the negative aspects of increasing environmental exposures. Film studies of cinematic trends have consistently recognized the thematic prevalence of “nuclear anxiety” in films from the 1950s and the plethora of films that depicted the horrendous “atomic mutations and mass devastation” resulting from nuclear exposure (Newman 2000). Films produced since the 1970s, in contrast, have focused on pollution more generally and the threats posed by toxic waste, contamination of the food chain and water supplies, and the increasing reality of diminishing resources (Frayling 2005). Unfortunately, cultural expressions about the outcomes of environmental pollution, as depicted in movies and books, have too often portrayed such scenarios in overly dramatic or unrealistic terms (Murray and Heumann 2014). Despite a few examples of positive outcomes (e.g., *A Civil Action*, *Silkwood*), the majority of cultural depictions of diminishing resources do not reflect optimism that science can “fix” pollution. Rather, the postapocalyptic film trend reflects a pervasive attitude that our current actions will lead to barbaric societies where diminishing resources have been completely depleted and climatological changes have spun out of control (e.g., *Mad Max*, *The Hunger Games*, *The Day After Tomorrow*).

The scientific community recognizes that media, and most recently social media, play a key role in public understanding of environmental risk (Fitzpatrick-Lewis et al. 2010; McCallum et al. 1991). Publications and news reports that are evidence based and reflect an understanding of science represent positive examples of media representation of environmental risks. However, the media can misrepresent environmental risks (and indeed have done so), tending to focus on the most dramatic aspects of exposure events and disasters, and presenting news about the outcomes of environmental health science research as a means of driving specific political agendas (Jaspal and Nerlich 2014a, 2014b). These information challenges must be considered as efforts are made to build EHL, especially when attempting to raise public understanding of actual versus perceived risks from environmental exposures.

Ultimately, evidence-based environmental health risk communications can help to provide more accurate evidence to counterbalance media and cultural representations of environmental degradation and its impact on human health. Furthermore, raising EHL can help individuals to navigate the abundance of information, of varying quality and veracity, that is available on the Internet (e.g., on-line blogs, chat rooms, other forms of social media) and can empower them to decide what choices are best for their health and that of their families (Wilcox 2012). More important, improving knowledge about environmental health risks can be used to promote a more optimistic view of the potential that exists to reduce, mitigate, or eliminate the worst environmental exposures and improve the health of both humans and the environment.

## EHL Methodology and Approaches

**Figure 2:**
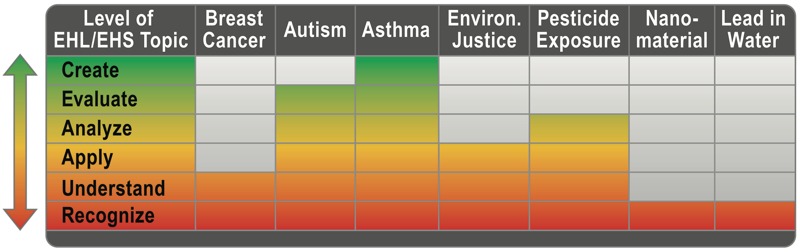
Conceptual model of environmental health literacy adapted from Bloom (1956), representing the potential for different levels of EHL across various environmental health science topics.

EHL builds on, synthesizes, and encompasses validated tools and methodologies from existing fields of research such as health literacy, risk communication, and education. Although the development of these approaches is most closely based on health literacy concepts and practices, several authors working in this emerging field conceptualize EHL as a process that individuals and communities embrace as a means of critical reflection within their local socioeconomic context rather than as a type of health literacy that incorporates specialized knowledge of environmental factors (Chinn 2011; Sykes et al. 2013). This concept of critical reflection was initially proposed by Nutbeam as one of three phases of learning and processing that reflect the evolutionary nature of health literacy (Nutbeam 2008). Although a number of articles cite this three-stage conceptual framework for EHL, we propose adapting Bloom’s taxonomy of educational objectives as a more nuanced model for the evolutionary nature of becoming more literate about environmental health issues (Bloom 1956) (see Figure 2).

Since its publication, more than 5,000 authors have found Bloom’s taxonomy to be a useful construct (Flinders 1996). Bloom’s stepwise progression of six distinct educational stages is a fitting approach for the development of targeted interventions for the various stages of EHL. The value of this model for describing the evolution of learning and understanding is that it acknowledges an individual’s potential for environmental health literacy at each stage. For example, those at the earliest stage, “Recognition,” know that a specific substance is toxic and may affect their health without any other understanding of how this occurs, what levels are concerning, or how to mitigate the exposure. This is, nonetheless, an initial stage of environmental health literacy. As the model suggests, the goal of EHL is to continue to promote greater understanding, to improve an individual’s extrapolation of knowledge to other potential environmental risks, and to stimulate actions based on the understanding of risk. However, the model is not meant to suggest a single path upward to total literacy or an equal level of literacy about different exposures; like the disease-specific nature of health literacy, an individual’s environmental health literacy may vary from topic to topic. For example, someone may have achieved a high degree of EHL related to asthma because of ongoing family experiences with this condition as well as the widespread public information linking asthman to air pollution, and yet possess a very low EHL regarding breast cancer and its lesser-known connections to environmental exposures.

The stages in the taxonomy also indicate the type of action individuals and communities might take based on their level of EHL. These actions can be wide-ranging, from an individual decision to avoid certain personal care products to a union movement to improve workplace conditions, each of which might represent a single stage of environmental health literacy. An example that represents all stages of this model could be a statewide movement to address potential health effects from hydraulic fracturing that builds from the recognition of the exposure to an extrapolation of a health risk to the creation of policy to address the risk. Individuals who are proficient in EHL are able to recognize their exposures and exert some manner of control over them rather than feeling as if “there’s nothing I can do.”

Environmental exposures most commonly affect communities as a whole; however, individual health outcomes arising from these exposures are dependent on an individual’s socioeconomic, biological, and psychological susceptibility to these exposures (Lee et al. 2005; Quandt et al. 2004). Therefore, efforts to promote EHL should include ways to measure literacy at individual and community levels as well as a range of information that recognizes the psychosocial and demographic heterogeneity within communities and the potential for distinct medical, psychological, or cultural responses to a common source of exposure(s). To be truly effective, efforts to promote EHL should be based on the types of awareness and knowledge needed, and they should use validated and culturally sensitive strategies to best promote the uptake of information by individuals, communities, public health officials, health care providers, or in regulatory or policy settings (Arcury et al. 2010; Ramos et al. 2001). An understanding of environmental health risks could serve as a needed mediator to improve media representations of environmental health science and in popular cultural representations of the relationship between the environment and health (Fitzpatrick-Lewis et al. 2010). More critically, raising EHL could be an important goal of science, technology, engineering, and math (STEM) educational efforts in vulnerable communities and could provide future generations with the knowledge, skills, and evidence to address environmental injustices that lead to health disparities.

## NIEHS Contributions to EHL

NIEHS has played an influential role in the emergence of EHL since the early 1990s. Since then, NIEHS programs have focused on building the capacity of researchers and community members to work together to address the environmental health concerns of community residents and related concerns about environmental justice and environmental health disparities. Although not specifically stated, these programs have shared a common goal: to build and strengthen EHL. To further this goal, NIEHS included community outreach, dissemination, translation, and education cores as required components of key programs (Hursh et al. 2011). Moreover, the institute transitioned from communication *to* the public to communicating *with* the public. One-way communication strategies changed to bidirectional and multidirectional approaches, including social media and other Internet-based modalities, to ensure that all partners could contribute to a dialogue about environmental health risks (Sullivan et al. 2003).

Community-engaged research (CEnR) programs at NIEHS have demonstrated how raising EHL can also serve as a tool for empowering individuals to actively participate in efforts to address environmental exposures of local or regional concern (Adams et al. 2011; Haynes et al. 2011). An additional positive consequence for promoting EHL is raising general scientific literacy and numeracy among the public.

Over time, these community-engaged programs fostered novel partnerships (Shepard et al. 2002), taught researchers how to work collaboratively with community residents (DeLemos et al. 2007), empowered community groups to be actively involved in the conduct and dissemination of research (Minkler et al. 2010), and trained teachers how to bring environmental health concepts into the classroom (Moreno and Tharp 1999). The NIEHS experience shows that cultivating equity in community–academic partnerships enables projects to develop effective and culturally appropriate materials for local communities. Additionally, sustained support for CEnR, which includes capacity building of all partners, allows projects to address environmental health disparities in vulnerable populations, such as Latino, Native American, African American, and low-socioeconomic-status communities. These programs have all addressed essential components of an EHL model that emphasizes the importance of health literacy for public health and prevention (Freedman et al. 2009; Sørensen et al. 2012). These successes, and the continued need to raise EHL and public health awareness of risks, have kept multidirectional communication and engagement as a central goal in the NIEHS 2012–2017 Strategic Plan (NIEHS 2012).

## EHL as a Research Topic

The trans-National Institutes of Health (NIH) Health Literacy program exemplifies NIH recognition of the need to explore fundamental issues in HL. For NIEHS, the focus is on validating effective ways of communicating about environmental health risks. Although the term EHL is increasingly used by investigators to denote a type of communications research, environmental health risk messaging is understudied, and relatively little is known about

whether there are specific stages of EHL that are amenable to intervention
whether raising EHL correlates with improved health outcomes
the relationship between EHL and resilience, for example, whether EHL increases the ability of an individual or a community to cope in challenging circumstances
the effectiveness of EHL resources and educational materials to inform intended audiences (within the context of their existing beliefs and attitudes)
different approaches for measuring success
the level of cultural acceptance of environmental risk messages in different ethnic and socioeconomic settings
the utilization and sustainability of evidence-based tools and approaches to raise EHL
whether risk messaging about environmental factors leads to behavior change
whether risk messaging leads to prevention, reduction or mitigation of environmental risk factors.

A key focus of EHL research will involve formal and rigorous assessment and validation to move from projects that produce new educational materials to projects that explore the effectiveness of educational resources. Additionally, research that explores EHL and advances the science of environmental risk messaging will require transdisciplinary or team science approaches. Environmental health scientists, individuals with expertise in community-engaged research, risk communication specialists, health educators, anthropologists, experts in dissemination and implementation science, community partners in research, and “citizen scientists” from affected communities will be critical to the success of this research.

## Conclusions and Recommendations

***Examine the influence of sociocultural context on EHL.*** When research focuses on ways to improve the EHL of individuals and communities, it will be important to understand the larger cultural context for how the public understands risks and to address misperceptions driven by media and cultural expressions. It is likely that media and films form the basis of beliefs and perception because they are widely accessed forms of communication and are often easier for the public to understand, rather than the more technical and scientific communication that investigators have historically disseminated. Effective efforts to raise EHL must therefore make risk messaging more understandable and more relevant to individuals, and they must provide not only the results of research but also address existing misinformation and misperceptions.

***Develop conceptual models.*** As EHL evolves, measuring its stages will be beneficial. We have modified Bloom’s taxonomy to enable targeted interventions for each stage of attainment in EHL. Our model should be tested and others developed or adapted, perhaps by utilizing or extending existing instruments from related fields to accurately measure and quantify the stages of EHL. Ideally, models should account for sociocultural context and how it influences EHL, and they should acknowledge skills and empowerment at each measurable stage of EHL.

***Use EHL as a tool for all partners.*** NIEHS embraces the evolution of EHL as an empowering component of community-engaged and environmental public health research. EHL research should include community partners in the research and provide capacity building and education at various levels of literacy for individuals and communities at risk from environmental exposures. Such education should extend beyond simply providing descriptions of specific risks to including some elucidation of the pace of science, the uncertainty principle, and the relevance of various risk measurements (e.g., ppb and levels of concern). Additionally, education and training of investigators in effective and appropriate communication modalities and creation of active partnerships with affected individuals will improve the development of culturally relevant messages. Health care professionals are another stakeholder group that could benefit from targeted education and training to enable them to recognize symptoms caused by environmental exposures and to diagnose environmentally induced diseases.

***Conduct EHL research.*** NIEHS is committed to advancing EHL, expanding on existing efforts, and addressing gaps in knowledge and practice. This commitment could include investigations to

characterize the process for increasing environmental health literacy
develop and validate measures of EHL at both individual and community levels
assess the effectiveness of existing environmental risk messages
measure the extent of behavior change based on health risk messaging
create or adapt environmental risk messaging to increase the EHL of specific audiences
identify statistical methods or develop models that correlate the role of EHL to improving the understanding of complex risk and health outcomes.

To be most effective, this research will require a transdisciplinary or team science approach, community–academic partnerships, and sufficiently broad expertise to allow development and dissemination of targeted messaging for local communities in modalities and languages that are culturally and linguistically appropriate. Special attention could be given to improving the EHL of low-literacy and non–English-speaking individuals or that of individuals living and working in health-disparate and low-income communities. Additionally, these projects should broaden the identification of relevant stakeholders and raise the EHL of not only affected community members but also that of health care professionals, public health and lay health workers, decision makers, teachers, and students.

***Coordinate federal resources.*** We recognize that NIEHS is only one player in the advancement of EHL and must work together with our federal partners such as the National Library of Medicine, the Centers for Disease Control and Prevention, the U.S. EPA, the National Science Foundation, and the Agency for Healthcare Research and Quality. As a coordinated group, representatives of these agencies could catalog and make available existing educational resources for the general public and for researchers working with chronically affected communities. Such a compilation of resources could provide a reliable, evidence-based source of information to the general public that may help to counteract the unsubstantiated (mis)information available on the Internet or disseminated through the media and films about environmental risks. This coordination will maximize the federal investments to date and help to ensure that research builds on previous efforts and utilizes effective tools and validated approaches developed in related fields.

Finally, the concept of EHL has emerged and is being embraced by investigators as a relevant research topic within environmental health sciences. We believe that the definitions and scope of EHL will continue to evolve and that research will help define the optimal approaches for measuring and raising EHL. Ultimately, efforts to improve EHL are intended to prevent environmentally induced disease and to empower individuals to gain a sense of control through understanding the environmental risks that affect their families and their communities.

## References

[r1] Adams C, Brown P, Morello-Frosch R, Brody JG, Rudel R, Zota A (2011). Disentangling the exposure experience: the roles of community context and report-back of environmental exposure data.. J Health Soc Behav.

[r2] Arcury TA, Estrada JM, Quandt SA (2010). Overcoming language and literacy barriers in safety and health training of agricultural workers.. J Agromedicine.

[r3] Barnes G, Fisher B, Postma J, Harnish K, Butterfield P, Hill W (2010). Incorporating environmental health into nursing practice: a case study on indoor air quality.. Pediatr Nurs.

[r4] Baur C (2010). New directions in research on public health and health literacy.. J Health Commun.

[r5] Benjamin R (2010). Improving health by improving health literacy.. Public Health Rep.

[r6] Biocca M (2004). Risk communication and the Precautionary Principle.. Int J Occup Med Environ Health.

[r7] Bloom BS (1956). Taxonomy of Educational Objectives: the Classification of Educational Goals. 1st ed..

[r8] Bonney R, Shirk J, Phillips T, Wiggins A, Ballard H, Miller-Rushing A (2014). Citizen science. Next steps for citizen science.. Science.

[r9] Brennan VM (2009). Natural Disasters and Public Health: Hurricanes Katrina, Rita, and Wilma..

[r10] Brown P Brody JG Morello-Frosch R Tovar J Zota AR Rudel RA 2012 Measuring the success of community science: the northern California Household Exposure Study. Environ Health Perspect 120 326 331, doi:10.1289/ehp.1103734 22147336 PMC3295345

[r11] Carson R, Darling L, Darling L (1962). Silent Spring..

[r12] Chan AH, Ng AW (2012). The guessing of mine safety signs meaning: effects of user factors and cognitive sign features.. Int J Occup Saf Ergon.

[r13] Chinn D (2011). Critical health literacy: a review and critical analysis.. Soc Sci Med.

[r14] Colucci-Gray L, Camino E, Barbiero G, Gray D (2006). From scientific literacy to sustainability literacy: an ecological framework for education.. Sci Educ.

[r15] Conrad CC, Hilchey KG (2011). A review of citizen science and community-based environmental monitoring: issues and opportunities.. Environ Monit Assess.

[r16] DeLemos J, Rock T, Brugge D, Slagowski N, Manning T, Lewis J (2007). Lessons from the Navajo: assistance with environmental data collection ensures cultural humility and data relevance.. Prog Community Health Partnersh.

[r17] Edwards JRD, Davey J, Armstrong K (2013). Returning to the roots of culture: a review and re-conceptualisation of safety culture.. Saf Sci.

[r18] Estacio EV (2013). Health literacy and community empowerment: it is more than just reading, writing and counting.. J Health Psychol.

[r19] Fitzpatrick-Lewis D Yost J Ciliska D Krishnaratne S 2010 Communication about environmental health risks: a systematic review. Environ Health 9 67, doi:10.1186/1476-069X-9-67 21040529 PMC2988771

[r20] Flinders DJ (1996). Bloom’s taxonomy: a forty-year retrospective [Book review].. Hist Educ Q.

[r21] Frayling C (2005). Mad, Bad and Dangerous? The Scientist and the Cinema..

[r22] Freedman DA, Bess KD, Tucker HA, Boyd DL, Tuchman AM, Wallston KA (2009). Public health literacy defined.. Am J Prev Med.

[r23] Gehle KS, Crawford JL, Hatcher MT (2011). Integrating environmental health into medical education.. Am J Prev Med.

[r24] Griswold E (2012). How ‘Silent Spring’ ignited the environmental movement. New York Times Magazine (New York) 21 September.. http://www.nytimes.com/2012/09/23/magazine/how-silent-spring-ignited-the-environmental-movement.html?_r=0.

[r25] Guidotti TL (2013). Communication models in environmental health.. J Health Commun.

[r26] Hancock HE, Rogers WA, Schroeder D, Fisk AD (2004). Safety symbol comprehension: effects of symbol type, familiarity, and age.. Hum Factors.

[r27] Hatfield TH (1994). A risk communication taxonomy for environmental-health.. J Environ Health.

[r28] Haynes EN, Beidler C, Wittberg R, Meloncon L, Parin M, Kopras EJ (2011). Developing a bidirectional academic-community partnership with an Appalachian-American community for environmental health research and risk communication.. Environ Health Perspect.

[r29] Hermer J, Hunt A (1996). Official graffiti of the everyday.. L & Soc Rev.

[r30] Huber JT, Shapiro RM, Gillaspy ML (2012). Top down versus bottom up: the social construction of the health literacy movement.. Libr Q.

[r31] Hursh DW, Martina CA, Trush MA, Davis HB (2011). Teaching Environmental Health to Children: An Interdisciplinary Approach..

[r32] IOM (Institute of Medicine of the National Academies) (2004). Health Literacy: a Prescription to End Confusion (Nielsen-Bohlman L, Panzer AM, Kindig DA, eds)..

[r33] IOM (2011). Innovations in Health Literacy Research: Workshop Summary..

[r34] Jaspal R, Nerlich B (2014a). Fracking in the UK press: threat dynamics in an unfolding debate.. Public Underst Sci.

[r35] Jaspal R, Nerlich B (2014b). When climate science became climate politics: British media representations of climate change in 1988.. Public Underst Sci.

[r36] Kaphingst KA, Kreuter MW, Casey C, Leme L, Thompson T, Cheng MR (2012). Health Literacy INDEX: development, reliability, and validity of a new tool for evaluating the health literacy demands of health information materials.. J Health Commun.

[r37] Kennedy MG, Turf EE, Wilson-Genderson M, Wells K, Huang GC, Beck V (2011). Effects of a television drama about environmental exposure to toxic substances.. Public Health Rep.

[r38] Lahr J, Kooistra L (2010). Environmental risk mapping of pollutants: state of the art and communication aspects.. Sci Total Environ.

[r39] Lee JEC, Lemyre L, Mercier P, Bouchard L, Krewski D (2005). Beyond the hazard: the role of beliefs in health risk perception.. Hum Ecol Risk Assess.

[r40] LePrevost CE, Storm JF, Blanchard MR, Asuaje CR, Cope WG (2013). Engaging Latino farmworkers in the development of symbols to improve pesticide safety and health education and risk communication.. J Immigr Minor Health.

[r41] Lesch MF, Rau PL, Zhao Z, Liu C (2009). A cross-cultural comparison of perceived hazard in response to warning components and configurations: US vs. China.. Appl Ergon.

[r42] Lin S, Gomez MI, Hwang SA, Franko EM, Bobier JK (2004). An evaluation of the asthma intervention of the New York State Healthy Neighborhoods Program.. J Asthma.

[r43] Matthews B, Andronaco R, Adams A (2014). Warning signs at beaches: do they work?. Saf Sci.

[r44] McCallum DB, Hammond SL, Covello VT (1991). Communicating about environmental risks: how the public uses and perceives information sources.. Health Educ Q.

[r45] Minkler M, Garcia AP, Williams J, LoPresti T, Lilly J (2010). Sí se puede: using participatory research to promote environmental justice in a Latino community in San Diego, California.. J Urban Health.

[r46] Minkler M, Vásquez VB, Tajik M, Petersen D (2008). Promoting environmental justice through community-based participatory research: the role of community and partnership capacity.. Health Educ Behav.

[r47] Mogford E, Gould L, Devoght A (2011). Teaching critical health literacy in the US as a means to action on the social determinants of health.. Health Promot Int.

[r48] Moore EE 2015 Green screen or smokescreen? Hollywood’s messages about nature and the environment. Environ Commun 1–17, doi:10.1080/17524032.2015.1014391

[r49] Moreno NP, Tharp BZ (1999). An interdisciplinary national program developed at Baylor to make science exciting for all K-5 students.. Acad Med.

[r50] Murphy MW, Iqbal S, Sanchez CA, Quinlisk MP (2010). Postdisaster health communication and information sources: the Iowa flood scenario.. Disaster Med Public Health Prep.

[r51] Murray RL, Heumann JK (2014). Film and Everyday Eco-disasters..

[r52] Newman K (2000). Apocalypse movies: end of the world cinema. 1st St. Martin’s Griffin ed..

[r53] Nicholson PJ (2000). Communicating occupational and environmental issues.. Occup Med (Lond).

[r54] NIEHS (National Institute of Environmental Health Sciences) (2012). Advancing Science, Improving Health: A Plan for Environmental Health Research. 2012-2017, Strategic Plan. NIH Publication No. 12-7935.. http://www.niehs.nih.gov/about/strategicplan/strategicplan2012_508.pdf.

[r55] Nutbeam D (2008). The evolving concept of health literacy.. Soc Sci Med.

[r56] Nutbeam D (2009). Defining and measuring health literacy: what can we learn from literacy studies?. Int J Public Health.

[r57] Paasche-Orlow MK, Riekert KA, Bilderback A, Chanmugam A, Hill P, Rand CS (2005). Tailored education may reduce health literacy disparities in asthma self-management.. Am J Respir Crit Care Med.

[r58] Perez H, Sullivan EC, Michael K, Harris R (2012). Fish consumption and advisory awareness among the Philadelphia Asian community: a pilot study.. J Environ Health.

[r59] Quandt SA Doran AM Rao P Hoppin JA Snively BM Arcury TA 2004 Reporting pesticide assessment results to farmworker families: development, implementation, and evaluation of a risk communication strategy. Environ Health Perspect 112 636 642, doi:10.1289/ehp.6754 15064174 PMC1241934

[r60] Ramos IN, May M, Ramos KS (2001). Environmental health training of promotoras in colonias along the Texas-Mexico border.. Am J Public Health.

[r61] Severtson DJ (2013). The influence of environmental hazard maps on risk beliefs, emotion, and health-related behavioral intentions.. Res Nurs Health.

[r62] Shepard PM Northridge ME Prakash S Stover G, eds. 2002 Advancing environmental justice through community-based participatory research. Environ Health Perspect 110(suppl 2) 139 140 11836141

[r63] Sørensen K Van den Broucke S Fullam J Doyle G Pelikan J Slonska Z 2012 Health literacy and public health: a systematic review and integration of definitions and models. BMC Public Health 12 80, doi:10.1186/1471-2458-12-80 22276600 PMC3292515

[r64] Stokes SC, Hood DB, Zokovitch J, Close FT (2010). Blueprint for communicating risk and preventing environmental injustice.. J Health Care Poor Underserved.

[r65] Sullivan J, Diamond PD, Kaplan CL, Mader TJ, Santa R, Lloyd RS (2003). Community outreach as an iterative dialogue among scientists and communities in the Texas gulf coast region.. Mutat Res.

[r66] Sykes S Wills J Rowlands G Popple K 2013 Understanding critical health literacy: a concept analysis. BMC Public Health 13 150, doi:10.1186/1471-2458-13-150 23419015 PMC3583748

[r67] Wilcox C (2012). Guest editorial. It’s time to e-volve: taking responsibility for science communication in a digital age.. Biol Bull.

[r68] Young SL (1998). Connotation of hazard for signal words and their associated panels.. Appl Ergon.

[r69] Zoller HM (2012). Communicating health: political risk narratives in an environmental health campaign.. J Appl Commun Res.

